# Stop Making Noise! Auditory Sensitivity in Adults with an Autism Spectrum Disorder Diagnosis: Physiological Habituation and Subjective Detection Thresholds

**DOI:** 10.1007/s10803-019-03890-9

**Published:** 2019-01-24

**Authors:** Marieke W. M. Kuiper, Elisabeth W. M. Verhoeven, Hilde M. Geurts

**Affiliations:** 1 Dr. Leo Kannerhuis, Autism Expert Centre, Department of Research, Development & Innovation, Houtsniplaan 1, 6865 XZ Doorwerth, The Netherlands; 2 University of Amsterdam, Faculty of Social and Behavioural Sciences, Dutch Autism & ADHD Research Center, Brain and Cognition, Nieuwe Achtergracht 129, 1001 NK Amsterdam, The Netherlands

**Keywords:** Habituation, Detection threshold, Autism, Auditory, Sensory sensitivity

## Abstract

Auditory sensitivities are common among people with autism spectrum disorder diagnoses (ASD). As underlying factors are unknown, we examined whether ASD adults (N_ASD_ = 33; N_Typically Developing_ = 31; 25–45 years; IQ > 70): (1) habituated slower to auditory stimuli; (2) had lower auditory detection thresholds; and (3) whether these mechanisms related to self-reported auditory sensitivities. Two auditory stimuli (tone, siren) were repeated, whilst skin conductance responses were recorded to measure habituation. Detection thresholds were measured by stepwise reductions in tone volume. We found no evidence in favor of our hypotheses, but ASD adults did rate the auditory stimuli as more arousing. Based on explorative analyses, we argue that studying the strength of physiological responses to auditory stimuli is needed to understand auditory sensitivities.

It is well known that many people with an autism spectrum disorder (ASD) diagnosis experience sensory sensitivities. Sensory sensitivities were already reported in the first descriptions of autism by Leo Kanner (Kanner 1943). Nowadays, sensory sensitivities are included as a criterion for the classification of ASD in the Diagnostic and Statistical Manual of Mental Disorders 5 (DSM-5; APA 2013). Even though someone can meet the criteria for an ASD classification without meeting the sensory sensitivity criterion, the reported prevalence of sensory sensitivities in people with ASD is high (60 to 96%; for review see Schauder and Benneto 2016). Besides this relatively high prevalence, sensory sensitivities are recently described to be related to other characteristics of ASD. For instance, sensory sensitivity has been related to social difficulties and the presence of more repetitive behavior (e.g., Deschrijver et al. 2017; for review see Jiujias et al. 2017). These findings make it even more crucial to examine possible underlying mechanisms of sensory sensitivity in ASD, as this might provide us with information we need to develop successful treatments for sensory sensitivities that are perceived as problematic by those with ASD. In the current paper, we will focus on two possible underlying factors that might play a role in auditory sensitivity in ASD adults, namely habituation and detection thresholds.

One of the most commonly reported sensory sensitivities in ASD is sensitivity to sounds (Baranek et al. 2006; Haesen et al. 2011; Jones et al. 2009; Kern et al. 2006; Kientz and Dunn 1997; Tomchek and Dunn 2007). Studies, clinical observations, and autobiographies show that people with ASD perceive certain sounds as more intense. For instance, certain frequencies can be extremely annoying (e.g., computer fan), loud noises can be painful (e.g., fog horn) and combined sounds such as multiple people talking to each other at once can be overwhelming (e.g., for review see Elwin et al. 2012; Robertson et al. 2015). Moreover, some ASD adults expressed that they were not able to get used to certain sensory stimuli as other people without ASD seemed to do (Robertson et al. 2015). This description is similar to what experimental studies on learning call “habituation”. Habituation refers to response distinction after a stimulus has repeatedly been presented (Houtveen et al. 2001). In other words, when a stimulus is repeated multiple times the physiological response to the stimulus slowly decreases or will get extinct. Habituation is an automatic form of learning in which the body learns not to physiologically respond to stimuli that are familiar, predictable or not relevant anymore (McDiarmid et al. 2017). The habituation description of people with ASD (Robertson et al. 2015) is in line with a hypothesis that states that some people with ASD might not or only slowly habituate to sensory stimuli (e.g., Hutt et al. 1964; Schoen et al. 2008; for review see; McDiarmid et al. 2017). These habituation difficulties to certain stimuli would lead to a “sensory overload” and hyper-reactions, which is commonly seen in people with ASD.

So far, studies on habituation in people with ASD show mixed results (for reviews see Lydon et al. 2016; McDiarmid et al. 2017). Studies use different measurements and stimuli to examine habituation, which might be a reason why mixed results are found (McDiarmid et al. 2017). For instance, habituation can be measured by determining the acoustic startle reflex and/or event-related potentials (ERP), by measuring electrodermal activity (EDA), or by means of functional magnetic resonance imaging (fMRI; McDiarmid et al. 2017). Studies using fMRI to study habituation in people with ASD have, so far, only focused on social stimuli (McDiarmid al. 2017). These studies (n = 5) showed that the amygdala of people with ASD habituated slower to faces compared to TD people (McDiarmid et al. 2017). Studies that have examined habituation to auditory stimuli (n = 7) focused mainly on children with ASD (Lydon et al. 2016) and all used EDA as measure for habituation. Results showed that children with ASD habituated either slower (e.g., Barry and James 1988; James and Barry 1984; Schoen et al. 2008; Stevens and Gruzelier 1984), or faster (e.g., Schoen et al. 2008), or there was no difference in habituation compared to a typical developing (TD) group (e.g., Chang et al. 2012; McCormick et al. 2014; van Engeland 1984). A study that both found slower and faster habituation in the ASD group (Schoen et al. 2008) suggested that it depended on the baseline skin conductance levels (SCL) of participants whether children with ASD habituated slower or faster. ASD children with high baseline SCL tended to habituate slower and ASD children with low baseline SCL tended to habituate faster. Baseline SCL is considered a proxy for sympathetic nerve activity (Dawson et al. 2007), with a higher SCL suggesting more physiological arousal. The only auditory habituation study that focused on adults showed that the ASD group did not differ from the TD group on habituation to a simple tone (e.g., Zahn et al. 1987). This study, however, had a small number of participants in each group (n_ASD_ =13, n_TD_ = 19, n_schizophrenia_ = 13). Given that auditory sensitivity persists into adulthood (e.g., Robertson et al. 2015), more knowledge on habituation in ASD adults is required.

Besides possible habituation abnormalities, lower auditory detection thresholds might also play a role in auditory sensitivities in people with ASD as they often report to hear sound sooner than TD people (e.g., Elwin et al. 2012; Talay-Ongan and; Wood 2000). Moreover, the enhanced perceptual functioning model (EPF) of Mottron and Burack (2001, 2006) suggests that in people with ASD information processing systems that are involved in detection, categorization, and discrimination of perceptual stimuli (a.k.a., visual and auditory stimuli) are enhanced (Mottron and Burack 2001; Mottron et al. 2006). This means that people with ASD will perform superior on tasks that are designed to measure these variables. Previous research showed indeed that adolescents and young adults with ASD performed superior compared to a TD group on an auditory discrimination and categorization task (e.g., Bonnel et al. 2003; Mottron et al. 2006). People with ASD also seemed to be faster in detecting a visual target and are more accurate in detecting hierarchical auditory stimuli (Mottron et al. 2006). There is also evidence for the opposite, namely that people with ASD are less able to detect a sensory stimulus. For instance, it is suggested that the more ASD traits one has, the higher their detection threshold is for tactile stimuli (e.g., Tavasolli et al. 2016). Also with regard to odor detection thresholds it seems that ASD children were less able to detect the stimuli than TD children did (Dudova et al. 2011). An auditory detection threshold refers to a minimum level of sound that is detectable for a person. Humans are able to perceive frequencies in the range from 20 to 20,000 Hz, and are most sensitive for auditory stimuli in the range from 2000 to 4000 Hz (Goldstein 2010), which is precisely the range that is important for understanding speech (Goldstein 2010). Sounds with an amplitude above 120–140 decibel (dB) are suggested to cause pain and be potentially damaging to the auditory system (Newman 1972). To our knowledge, studies on auditory detection thresholds in people with ASD are scarce. One small study showed that 11 ASD children did not differ from 11 children without ASD on auditory detection thresholds regardless of frequency (i.e., 0.25 kHz, 0.5 kHz, 1 kHz, 4 kHz and 8 kHz; Khalfa et al. 2004). In another small study, with only 12 young adults in each group, it was mentioned that the ASD participants did not have lower auditory thresholds compared to the TD participants (Bonnel et al. 2003). Therefore, in the current study, we will include a much larger ASD adult sample while we follow the method of Khalfa et al. (2004).

In this study, we will test three hypotheses. We hypothesize that (1) ASD adults habituate slower than TD adults; (2) ASD adults have lower auditory detection thresholds than TD adults; and (3) habituation and auditory detection threshold are underlying factors of the often reported auditory sensitivities. Based on this third hypothesis, we expect a negative correlation of habituation as well as auditory detection threshold with self-reported auditory sensitivity. Additionally, we explored the hypothesis of Schoen et al. (2008) that baseline arousal [as indicated by baseline SCL and heart rate variability (HRV)] is related to habituation and auditory detection thresholds.

## Methods

### Participants

ASD participants were recruited at specialized clinical centers for people with ASD and through advertisements on several websites (e.g., website of the Dutch association of Autism). To be included, ASD participants needed to have a clinical DSM-IV or DSM-5 ASD diagnosis (i.e., DSM-IV Asperger Syndrome, PDD-NOS, Autism, or DSM-5 Autism Spectrum Disorder), diagnosed by a clinician specialized in ASD prior to enrolment in this study. To be included in the ASD-group, they also needed to score above the cut-off of 54 on the Social Responsiveness Scale—Adults (SRS-A; Constantino and Gruber 2005; Dutch version:; De la Marche et al. 2009) or score above the cut-off of 26 on the Autism Quotient (AQ; Baron-Cohen et al. 2001; Dutch version:; Hoekstra et al. 2008). To describe the ASD features of our ASD group, we administered the Autism Diagnostic Observation Schedule 2 (ADOS 2; Hus and Lord 2014; Lord et al. 2012), module 4 (see Table 1). In the ASD group, 42.4% used psychotropic medication. The most common used medications were antidepressants (n = 10) and antipsychotic medication (n = 5). TD participants were recruited through the personal network of the researchers and students involved in this project and matched closely on age and gender with the ASD group. Inclusion criteria for the TD group were (1) having no (suspicion of a) clinical diagnosis of ASD or any other psychiatric or developmental disorder; (2) having no immediate family member (e.g., brother, sister, father, mother) with ASD or psychotic disorder; (3) scoring below the cut-off of 54 on the SRS-A; (4) scoring below the cut-off of 26 on the AQ; and (5) no psychotropic medication use.

Criteria for both the ASD and TD group were: (1) being between 25 and 45 years of age; (2) having an estimated IQ score ≥ 70 on the abbreviated Wechsler Adult Intelligence Scale IV (WAIS-IV; Wechsler 2012; i.e., Matrix Reasoning and Vocabulary; Uterwijk 2000); (3) having no neurological diseases or epilepsy; (4) having no heart disease; (5) having no lung disease (e.g., Asthma); (6) use no beta-blocker medication; and 6) have no known hearing impairment (e.g., hearing loss or Tinnitus).

**Table 1:** Group descriptives and statistics

	ASD (n = 33)	TD (n = 31)	p-value	Cohen’s d
Gender	17M; 16F	16M; 15F	–	–
Descriptives				
Age (years)	33.70 (5.4)	33.74 (6.3)	0.98	0.01
TIQ	104.30 (16.8)	103.74 (16.0)	0.89	− 0.03
AQ	35.52 (6.4)	10.35 (4.9)	0.00***	− 4.40
SRS-A	97.18 (21.8)	22.42 (11.2)	0.00***	− 4.28
ADOS				
ADOS-2 (mod.4) total	8.39 (5.1)	–	–	–
ADOS-2 (mod.4) SA	7.36 (4.3)	–	–	–
ADOS-2 (mod.4) RRB	1.03 (1.5)	–	–	–
AASP				
Auditory total score	35.61 (8.1)	24.94 (6.0)	0.00***	− 1.49

We used a Bonferroni correction to compensate for the multiple comparisons (Bonferroni correction: α = 0.05/5 = 0.01).

*AASP* adolescent/adult sensory profile, *ADOS-2 (mod. 4)* autism diagnostic observation schedule (2) module 4 total score (≥ 8 classifies as autism spectrum; Hus and Lord 2014), *ADOS SA* social affect, *ADOS RRB* restricted repetitive behavior, *ASD* autism spectrum disorders, *AQ* autism quotient, *Cohen’s d* effect size (small = 0.20; medium = 0.50; large = 0.80), *F* female, *M* man, *n* number of participants, *SRS-A* social responsiveness scale-adults, *TD* typical developing, *TIQ* total intelligence quotient

***p ≤ 0.001

### Materials

#### Habituation Task

The habituation task is a computerized task were two different auditory stimuli were presented to the participants over headphones for 3 s (iMG Stage line MD-5000DR). Each of these auditory stimuli were presented 15 times in separate blocks within the task (i.e., condition 1 and 2). Condition 1 consisted of a 1000 Hz simple tone (84 dB; see for a similar approach Schaaf et al. 2015). In condition 2 the presented auditory stimulus was a Dutch ambulance siren (78 dB; Schaaf et al. 2015). In both conditions, the interstimulus interval was jittered between 20 and 35 s, which is in line with the recommendations for electrodermal measurements (Boucsein et al. 2012).

As in earlier studies (e.g., Boucsein et al. 2012; Chang et al. 2012; Rothbaum et al. 2001), habituation was defined as two consecutive trials on which no skin conductance response (SCR) occurs. If a SCR amplitude reached above 0.03 microsiemens (µS) and the SCR occurred within 1–4 s after a stimulus was presented (Boucsein et al. 2012), it was counted as a SCR. SCR was measured with two curved Ag/AgCl electrodes (20 by 16 mm) and calculated in VSRRP98 by analyzing the first derivative of the signal where the algorithm searches for peaks and troughs (changes in first derivative sign) in the signal. The VSSRP98 is a software developed by the University of Amsterdam and used in several published articles (e.g., Bos et al. 2013; Krypotos et al. 2011; Kuiper et al. 2017; van Well et al. 2012). The signal is low-pass filtered at 10 Hz, 4th order Butterworth, before applying the algorithm. After identifying the peaks in the signal, the algorithm searches backward for troughs and bends, during a given interval (4000 ms). When a so called peak/trough pair is found, the algorithm applies user given criteria to decide whether a marked peak is valid or not. In the current study, the user given criteria are an amplitude of at least 0.03 µS with a response window of at least 1000 ms and at most 4000 ms after the stimulus was presented.

The dependent measure for habituation was the number of trials participants needed to habituate. This is also referred to as “the completion process of habituation” (Boucsein et al. 2012). For instance, if a person responded with a SCR to the first four trails and did not show a SCR on trial five and six, then four was the number that was recorded for the habituation analysis. Participants who showed no SCR above 0.03 µS within 1–4 s after the stimulus on one of the first two trials (siren or simple tone) were considered non-responders and were left out of the habituation analysis (e.g., Schoen et al. 2009; Iacono et al. 1999; Ohman et al. 1989). The number of non-responders per group is reported in the “Results” section. Please note that all of the above definitions and criteria were included in our ethics approval of the University of Amsterdam, before the current study started. However, as in the literature habituation is sometimes also defined as decreasing SCR magnitudes to repeated stimuli (e.g., Boucsein et al. 2012), we chose to explore this habituation definition as well.

#### Auditory Detection Task

Auditory detection thresholds were measured similarly to the method described in the study by Khalfa et al. (2004). The auditory stimulus (a simple tone, 1000 Hz) was set at 60 dB HL and was decreased in steps of 5-dB until the participants verbally reported they could no longer hear the tone. On a laptop screen, the participant saw a green cross which turned white when a tone was presented, so they had a cue for knowing when a response was required. Participants needed to say “yes” if they could still hear the tone and say “no” when they did not hear the tone anymore. When participants said “no”, the tone was increased in 5-dB steps until the participant verbally reported they could hear the tone again. The lowest intensity at which the tone was perceived will correspond to the threshold. This procedure was repeated a second time. If the values were different, it was repeated until two consecutive times the detection threshold was the same. More than two repetitions were needed for 39.4% of the ASD and 26.7% of the TD participants to reach the same detection threshold two consecutive times. The simple tone was presented for 500 ms each time, with a onset/offset ramp of 50 ms. The dependent measure is the lowest number of dB that is detectable for the participant.

#### Subjective Ratings Auditory Stimuli

After both auditory stimuli were presented in the habituation task, participants were asked to rate both stimuli on valence and arousal. The self-assessment manikin procedure (Lang et al. 2005; Bradley and Lang 1994; Kuiper et al. 2017) was used. The first question regarded valence: “How happy/not happy did you feel when you heard the tone?”. Participants answered this question by giving a rating between 1 (not happy) and 9 (very happy). The second question regarded the level of arousal: “How excited/calm did you feel when you heard the tone?”. Again, participants provided a rating between 1 (very calm) to 9 (highly stressed). These questions were repeated for the siren.

#### Self-reported Auditory Sensitivity

Self-reported auditory sensitivity was measured with the Adolescent Adult Sensory Profile (AASP; Brown and Dunn 2002; Dutch version:; Rietman 2007). The AASP is a 60-item questionnaire. Each of these items described sensory related behaviors and experiences such as “not noticing when your name is called”. Participants could answer these items on a 5-point scale, with answer possibilities ranging from “almost never” to “almost always”. In this study, we used the auditory items (e.g., Jones et al. 2009) to calculate self-reported auditory sensitivity by adding up the scores of the auditory items (n = 10; items 50–60). A higher score means that this sensory behavior or experience occurs more often.

#### Baseline HRV and SCL

In order to explore whether arousal, indicated by baseline HRV and SCL, might be related to habituation rate and subjective auditory detection thresholds, we measured HRV by means of an electrocardiograph (ECG) and three Ag/AgCl electrodes (3M Red Dot Electrodes). The signal was analyzed by the VSRRP98 (version 10.1) as well. We used a frequency domain measure for HRV, namely respiratory sinus arrhythmia (RSA). RSA was quantified according to the “Porges Method” (Porges et al. 2013). For more detailed information on how we measured and calculated RSA, see the study of Kuiper et al. (2017) where a similar method was used to address a different research question. To check whether all participants fell within the breathing rate range, we also measured breathing rate. SCL is calculated with the VSSRP98 by means of averaging all samples (1000S/s) for the last 5 min of the 10 min baseline period. There was no need to low-pass the signal before averaging, because the process of averaging is a low-pass filter in itself.

### Procedure

Via mail, participants received general information regarding the procedures at the test-session and also questionnaires that needed to be answered before the test-session. At the actual test-session, a more in-depth explanation of the general test-session was given, which was followed by the placement of the ECG electrodes. One electrode was placed on the left side of the chest on the ribs, one below the right clavicle and the reference (ground) electrode was placed below the left clavicle. Breathing rate was measured with a respiration belt, which was placed just below the ribs and around the chest. The electrodes for the skin conductance measures were placed on the index and ring finger of the left hand of participants. Participants were instructed to sit calmly and quietly in the chair, as movement could influence the signal. During the baseline period, which lasted 10 min, participants sat calmly in a chair and tried to relax.

After the baseline period, all participants did the habituation task as we wanted to avoid that other tasks (which also included tones or sounds) could influence the habituation measurement. After the habituation task, the order in which the other measures were presented was randomized. The tasks were: the detection threshold task, the ADOS, the two subtests of the WAIS-IV and a computerized visual short-term memory task (similar to the task used in Pinto et al. 2013). After three months participants received follow-up questionnaires at home, which they could return by mail. The visual short-term memory task as well as the follow up questionnaires are not part of this particular study and will, therefore, be reported elsewhere.

Participants received between 10 and 15 euros for participating and up to 20 euros for travel expenses, depending on the costs they made.

### Statistical Analyses

Our power estimation regarding habituation was based on a study of James and Barry (1984) and a study of Schaaf et al. (2015). The reported effect size for group differences (ASD vs. TD) with regard to habituation or sympathetic activity in response to an auditory stimulus was respectively 3.34 and − 1.02 (Cohen’s d). Both fell within the range of 0.8 and higher, which is considered a large effect size (Cohen 1988). In our power analysis (based on a MANOVA, between subject design) we used the lower bound of a large effect size. The power analysis was performed using the program G*Power (Faul et al. 2009). In G*power, one needs to enter an effect size f (not d) and the lower bound of a large effect size f is 0.4. We, therefore, entered the following numbers: effect size f 0.4; 2 groups (ASD vs. TD), number of measures 2 (tone and siren habituation) and a large expected correlation of 0.8 between the measures as both are auditory stimuli presented in a similar fashion. The power analysis showed that we needed a total sample of our study of 48, so 24 participants in each group. As mentioned at ‘*material’*, it is possible some adults are “non-responders”. The non-responders cannot be not included in the habituation analyses and, therefore, we continued to recruit and test participants until we had 24 “responders” per group for the habituation analyses. The number of non-responders and participants that needed to be excluded from the habituation analyses due to technical artifacts are mentioned in the “Results” section.

Before running the analyses, we checked whether the variables were normally distributed among the groups, by calculating Skewness and Kurtosis, and transforming them into Z-scores (Field 2009). This showed that the AQ, baseline RSA, baseline SCL, detection threshold and the siren-valence variables were not normally distributed. After a log transformation for the detection threshold variable the data was normally distributed. The siren-valence variable was not transformed as it is an ordinal scale that is not a true ratio scale. This is also the case for the other subjective rating variables (i.e., siren-arousal, tone-valence and tone-arousal). We used a non-parametric test for these specific variables. For the other variables, transforming the data did not result in a normal distributed variable. We also calculated whether the included variables contained outliers. Outliers are defined as data points more than three times the interquartile range above or below the first quartile. Outliers were only detected in the AQ variable (1 ASD), in the baseline RSA variable (3 ASD; 1 TD) and in the baseline SCL data (2 ASD). In accordance with Field (2009), we gave the outliers the value of the next highest or lowest data point plus one “unit”. After this procedure, the data of all three measures normally distributed.

Our analyses can be subcategorized into three parts. Part 1 (group descriptives): we ran one-way ANOVA’s to examine whether the two groups (ASD vs. TD) differed on age, TIQ, AQ, SRS-A, Baseline RSA, Baseline SCL and AASP auditory items. We used Bonferroni corrections to compensate for the multiple comparisons (please see subscript of each Table).

Part 2 (main analyses): for our main (i.e., confirmatory) analyses, we first examined whether habituation in response to auditory stimuli differed between the ASD and the TD group, we conducted a 2 × 2 MAVOVA with habituation (tone, siren) as within subject factor and group (ASD, TD) as between subject factor. Second, we examined whether self-reported auditory sensitivity was related to habituation rate, a Pearson correlation was calculated between habituation (tone) and the total score of the auditory items of the AASP. Third, we examined whether detection thresholds in response to auditory stimuli differed between the ASD and the TD group, we conducted an one-way ANOVA with the number of dB at which a participant still can detect the sound as dependent variable and “group” (ASD vs. TD) was between subject factor. Fourth, we examined whether self-reported auditory sensitivity was related to auditory detection threshold, a Pearson correlation was calculated between detection threshold and the total score of the auditory items of the AASP. Both the habituation and detection analyses were repeated with ‘medication-use’ as covariate to explore whether any medication use of the ASD group has affected the main pattern of findings. Fifth, we examined whether the subjective ratings of the stimuli used in the current study (tone and siren) were related to self-reported auditory sensitivity. So, we calculated Spearman correlations between the subjective ratings variables (i.e., siren-valence; siren-arousal; tone-valence; tone-arousal) and self-reported auditory sensitivity (AASP auditory items).

Part 3 (explorative analyses): in the additional explorative analyses, we ran Pearson’s correlations between habituation rate (tone) and baseline arousal (i.e., SCL and HRV) to explore the hypothesis of Schoen et al. (2008) that baseline arousal would be positive related to habituation and explored this for auditory detection threshold as well. Second, as we observed (see “Results” section) that the two groups (ASD vs. TD) did not differ from each other on habituation in our main analysis, we explored habituation by means of another common habituation definition, namely by exploring the decrease in SCR magnitude across the 15 trials. To reduce our potential power problem for these analyses, we created 3 blocks of 5 trials each, which were used to explore the SCR slope. We calculated the mean SCR magnitude per block for each group. Then we performed a repeated measures ANOVA (23 ASD; 22 TD) with “Stimulus Block” as within subject factor (3 levels) and “group” as between subject factor (2 levels). We repeated this for the siren stimulus.

Besides the conventional statistical analyses, we added Bayesian statistics to assess how much evidence there is for the “alternative” hypothesis (H_a_) over the “null” hypothesis (H_0_). H_a_ represents our hypotheses that there are group differences and H_0_ refers to the hypothesis that both groups are the same. We will report the Bayes Factor 10 (BF10), which represents the likelihood that H_a_ is true relative to H_0_, given our data. In simpler terms, a higher BF10 indicates that there is more evidence for group differences. We used the program JASP (JASP Team 2017; Love et al. 2015; Morey et al. 2015) to run the Bayesian (Repeated Measures) ANOVA’s. We also used the program Statcheck (Epskamp and Nuijten 2016) to check whether all our reported *p*-values are correctly reported.

## Results

### Part 1: Group Descriptives

#### Participants

In total, 64 participants (33 ASD, 31 TD) were originally included in this study. Due to a technical problem with some of the skin conductance electrodes, the physiological data of five participants (2 ASD; 3 TD) could not be used. Therefore, these participants were not included in the habituation analyses. Both groups also had a number of non-responders, namely 21.2% of the ASD group (n = 7) and 12.9% of the TD group (n = 4). Those non-responders were not included in the habituation analyses as well. This means that in the habituation analyses both groups consisted of 24 participants each. In the other analyses, all 64 participants were included.Before we ran our analyses, we examined by means of one-way ANOVA’s (Bonferroni correction: α = 0.05/11 = 0.0045) whether the non-responders differed from the responders of the same group (ASD or TD) on several variables, namely age, TIQ, AQ, SRS-A, ADOS-SA, ADOS-RRB, ADOS-total, baseline RSA, baseline SCL, baseline HR, detection threshold (see Table 3). The ASD non-responders scored significantly higher compared to the ASD responders on ADOS social affect (SA; *F*(1,30) = 12.45; *p* = 0.001; Cohen’s d = 1.52) and total ADOS score (*F*(1,30) = 9.75; *p* = 0.004; Cohen’s d = 1.34). All other ASD or TD comparisons were non-significant (*p-range* = 0.054 − 0.991; Cohen’s *d* range = 0.08–1.05).

#### Group Differences

The ASD group and TD group did not significantly differ on age and TIQ (for descriptives and statistics see Table 1). However, as expected the ASD adults reported more auditory sensitivities (AASP auditory items). Moreover, the ASD group did have a significantly higher baseline heart rate (HR).

### Part 2: Main Analyses

#### Auditory Detection Threshold

**Fig. 1:**
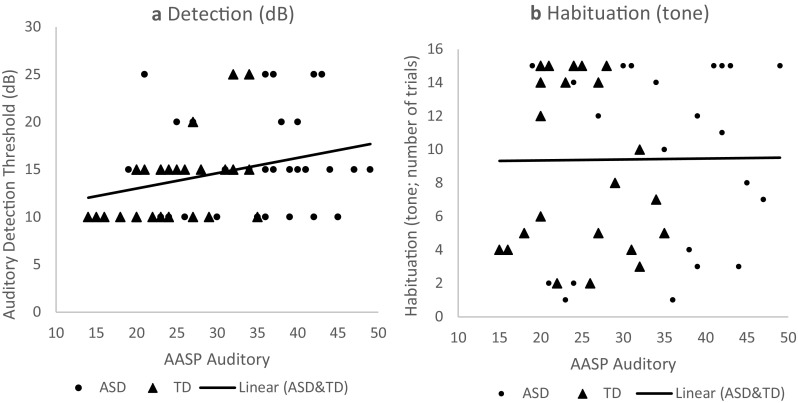
Pearson’s correlation of self-reported auditory sensitivity with detection threshold (left) and habituation (right)

The ASD group did not significantly differ from the TD group with regard to auditory detection threshold (for statistics see Table 2). BF10 was 0.52, which indicates that there is little to no evidence for H_a_ (i.e., group differences). Adding medication use as covariate did not change the results. There was a medium statistically significant positive correlation between auditory detection threshold and self-reported auditory sensitivity (*r* = 0.31, *p* = 0.01; see Fig. 1a). BF_10_ was 2.94, which indicates that there is slightly more evidence for H_a_. However, please note that instead of the expected negative correlation, we observed a positive correlation. Thus, adults with a higher auditory detection threshold reported more auditory sensitivities.

**Table 2:** Statistics of detection, habituation, and subjective ratings

Detection (dB)	ASD (n = 33)	TD (n = 31)	F value	p value	Cohen’s d
Tone	15.45 (5.2)	13.87 (4.6)	1.67	0.20	− 0.32

Habituation (trials)	ASD (n = 24)	TD (n = 24)	F value	p value	Cohen’s d
Tone	9.42 (5.6)	9.33 (5.1)	0.00	0.96	− 0.02
Siren	7.54 (5.6)	7.38 (5.7)	0.01	0.92	− 0.03

Subjective ratings	ASD (n = 33)	TD (n = 31)	F value	p value	Cohen’s d
Valence					
Tone	2.02 (0.1)	2.14 (0.1)	3.23	0.08	1.74
Siren	4.09 (0.3)	4.73 (0.3)	1.02	0.32	0.31
Arousal					
Tone	5.27 (0.3)	3.47 (0.3)	14.75	0.00***	5.44
Siren	4.97 (0.3)	3.57 (0.4)	8.56	0.01**	4.14

*ASD* autism spectrum disorders, *Cohen’s d* effect size (small = 0.20; medium = 0.50; large = 0.80), *dB* decibel, *n* number of participants; siren: the analyses were done with a square root transformed variable, *TD* typical developing; trials = number of trials. We used a Bonferroni correction to compensate for the multiple comparisons of the subjective ratings analyses (Bonferroni correction: α = 0.05/4 = 0.013)

**p ≤ 0.01, ***p ≤ 0.001

#### Habituation

No significant group differences on habituation were observed in response to both the tone and the siren (again, see Table 2). BF10 was 0.32 for the tone and 0.28 for the siren, which indicates there is little to no evidence for H_a_ (i.e., group differences). Adding medication use as a covariate did not change the results. In contrast to our hypothesis, there was no significant positive correlation between habituation rate and self-reported auditory sensitivity (*r* = 0.02; *p* = 0.90; see Fig. 1b). BF10 was 0.18, which indicates there is little to no evidence for H_a_ (i.e., correlation). However, the ASD group rated the tone and the siren as significantly more arousing than the TD group (tone: BF10 = 87.16; siren: BF10 = 8.41), but did not significantly differ in the valence ratings (for statistics see Table 2; tone: BF10 = 0.99; siren: BF10 = 0.40).

We also examined whether the valence and arousal ratings are related to self-reported auditory sensitivity (Bonferroni correction: α = 0.05/4 = 0.013). The arousal ratings of both auditory stimuli were positively correlated to self-reported auditory sensitivity (n = 63; tone: *r*_*s*_ = 0.61; *p* < 0.001; siren: *r*_*s*_ = 0.49; *p* < .001). The valence ratings of both the tone and siren were negatively correlated to the self-reported auditory sensitivity (tone: n = 63; *r*_*s*_ = − 0.44; *p* < .0.001; BF10 = 77.73; siren: n = 63; *r*_*s*_ = − 0.32; *p* = .0.012). We could not calculate a Bayes Factor for the Spearman correlations as this analysis is not yet available in JASP.

### Part 3: Explorative Analyses

Across all participants, the four Pearson’s correlations between baseline RSA and SCL (for statistics see Table 3) and habituation and detection threshold were non-significant (*r* = .03 − 0.17; *p* = .0.19 − 0.85).

**Fig. 2:**
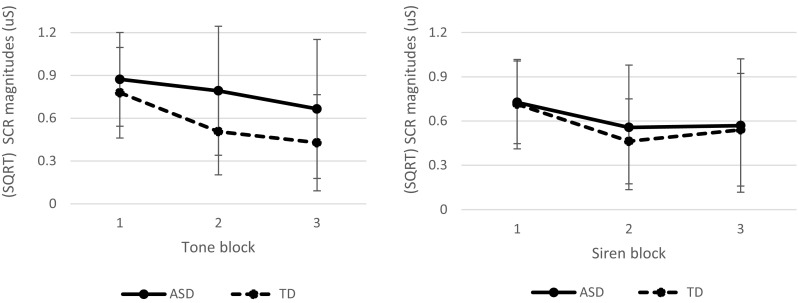
Mean (SQRT) SCR magnitude slope for each group and per stimulus: tone (left) and siren (right). Each block contains five repetitions. Both groups showed a decrease in SCR magnitudes in response to both stimuli. The ASD group had a significant higher SCR magnitude overall in response to the tone compared to the TD group

We also explored whether our choice for operationalizing habituation might have influenced our findings by analyzing whether both groups differed from each other on SCR magnitude. The blocks were not normally distributed. After calculating and removing the outliers (1 ASD; 2 TD), a square root transformation was required to achieve a normal distribution. Analyses regarding the tone showed that the groups significantly differed from each other on overall SCR magnitude, independent of type of block (*F*(1,43) = 4.44, *p* = 0.04, Cohen’s *d* = 0.64; see Fig. 2a). However, BF10 was 0.80, which means that there is little to no evidence for H_a_ (i.e., group differences). Moreover, there was no significant interaction between tone block and group (*F*(2,86) = 2.04, *p* = 0.14, Cohen’s *d* = 0.44). As typically observed, the SCRs magnitudes did decrease overall with each following block (*F*(2,86) = 16.36, *p* < 0.001, Cohen’s *d* = 1.23). Regarding the siren, no large differences between the groups was observed on overall SCR magnitude *F*(1,43) = 0.24, *p* = 0.63, Cohen’s *d* = 0.15; see Fig. 2b). BF10 was 0.422, which indicates there is little to no evidence for H_a_. There was no significant interaction as well (*F*(2,86) = 0.40, *p* = 0.67, Cohen’s *d* = 0.01). Again, as typically observered, the SCRs magnitudes decreased overall with each following block (*F*(2,86) = 10.32, *p* < 0.001, Cohen’s *d* = 0.98).

**Table 3:** Baseline arousal statistics

	ASD (n = 32)	TD (n = 31)	*p*-value	Cohen’s d
Baseline				
RSA (In(ms^2^))	6.28 (1.2)	6.13 (1.3)	0.62	− 0.13
SCL (uS)	32.31 (4.2)	31.16 (3.0)	0.22	− 0.14
HR	75.43 (9.7)	69.09 (9.4)	0.01**	− 0.89

We used a Bonferroni correction to compensate for the multiple comparisons (Bonferroni correction: α = 0.05/3 = 0.02).

*ASD* autism spectrum disorders, *Cohen’s d* effect size (small = 0.20; medium = 0.50; large = 0.80), *HR* heart rate, *M* man, *RSA* respiratory sinus arrhythmia, *SCL* skin conductance level, *TD* typical developing

**p ≤ 0.01

## Discussion

This study aimed to explore two possible underlying mechanisms of auditory sensitivity in ASD adults, respectively habituation and detection threshold. Contrary to our expectations, our results indicate that habituation as well as auditory detection threshold do not seem to play a role in auditory sensitivity in ASD adults. No group differences in the habituation and detection task were found. Moreover, we found no substantial evidence for a relationship of habituation and detection threshold with self-reported auditory sensitivity. Nonetheless, ASD adults reported more auditory sensitivity on a self-report questionnaire and also subjectively rated the auditory stimuli used in this study as more arousing than the TD adults. Also, our explorative analysis hinted towards the possibility that ASD adults might also physiologically respond more intense to certain auditory stimuli than TD adults as they overall showed higher SCR magnitudes to the presented tone (but habituated similarly). This should be interpreted with caution, because the Bayes Factor showed there was little evidence for this hypothesis. Our results do suggest that both habituation processes and detection threshold do not account for this heightened subjective and the potentially heightened physiological response.

Our confirmative and explorative habituation findings are in line with earlier studies in ASD children (e.g., Chang et al. 2012; McCormick et al. 2014; van Engeland 1984). Methodologically, these studies are similar to ours, namely (1) these studies also included either a simple tone (van Engeland 1984) or a siren (McCormick et al. 2014) or both a tone and siren (Chang et al. 2012); (2) defined habituation either as decreasing SCR magnitudes (McCormick et al. 2014) or number of trials until on two or three consecutive trials no SCR is given (Chang et al. 2012; van Engeland 1984); and (3) the volume of these studies were similar to ours (84 dB tone and 78 dB siren; Chang et al. 2012; 95 dB siren; McCormick et al. 2014; 85 dB tone; Van Engeland 1984). IQ scores were not reported in two of these studies (Chang et al. 2012; McCormick et al. 2014), but the mean IQ was above 70 in the study of Van Engeland (1984). However, there are also three studies that did report habituation differences (Barry and James 1988; James and Barry 1984; Stevens and Gruzelier 1984). These studies differ from the current study in three domains, namely (1) the IQ of the ASD participants (all mean IQ < 60); (2) the method chosen to measure habituation (e.g., respiratory pause; e.g., Barry and James 1988; James and Barry 1984); and (3) potentially the so called severity of ASD. To start with the latter, about 60% of our total ASD group did not met the criteria for an ADOS diagnosis of ASD (total score ≥8; Hus and Lord 2014), even though all of them had a clinical ASD diagnosis. This could mean that our ASD group perhaps had a milder form of ASD. Regarding the differences in IQ, it remains unclear whether or not intellectual disability (IQ < 70) plays a role in habituation, although a recent study on habituation of event-related potentials to auditory stimuli did show that individuals with Fragile X syndrome (a neurodevelopmental disorder, which is associated with intellectual disability; Chonchaiya et al. 2009) habituated more slowly to a simple 1000 Hz tone (Ethridge et al. 2016). Taken together, these differences in methodology and participant characteristics between the studies might explain (part of) the difference in findings.

Like in our study, two earlier, albeit rather small, studies did also report a lack of difference between people with and without ASD in auditory detection threshold (Bonnel et al. 2003; Khalfa et al. 2004). This suggests, combined with the observed low effect size and small Bayes Factor in the current study, that the observed auditory sensitivities in ASD people are not explained by a low auditory detection threshold, even though there was a small positive relationship between detection threshold and self-reported auditory sensitivity. The Bayes Factor for the latter relationship indicated that there was little to no evidence for this relationship, which means that there is not sufficient evidence to overrule our aforementioned conclusion. However, one could argue that instead of focusing on a sole frequency we should have included multiple frequencies to determine auditory detection thresholds before we can draw such a strong conclusion. Though, Khalfa et al. (2004) tested already a wide range of frequencies and did not observe any differences either. So even though adults with ASD often report to hear sounds sooner than TD people (e.g., Elwin et al. 2012), it seems that there is no actual difference in auditory detection threshold.

Our explorative finding that ASD adults showed a larger overall SCR magnitude to the presented tones is in line with studies that also found stronger sympathetic responses to auditory stimuli in children with ASD (e.g., Chang et al. 2012; Schaaf et al. 2015). SCR magnitudes resemble sympathetic activity responses (Dawson et al. 2007), in this case to the auditory stimuli. Larger SCR magnitudes suggest that ASD adults might physiologically respond more strongly, more intense. While we did not initially focus on the strength of the SCR to the auditory stimuli, this could well be an interesting future research avenue to pursue. Our observation that ASD adults respond physiologically stronger (i.e., larger SCR magnitude) to simple tones needs to be replicated, but it does make sense as the ASD adults subjectively experienced the tone as more arousing than the TD adults as well. However, ASD adults also subjectively experienced the siren as more arousing than TD adults, but there seemed to be no difference between the groups in intensity of the physiological response to the siren. This could suggest that perhaps other factors might be related to whether or not ASD adults respond physiologically stronger. A possible factor that might play a role is the volume of the auditory stimulus as the tone was presented louder than the siren. Some might argue that another possible explanation could be related to epigenetic determinism, which indicates that underlying genetics may be the same but the expression (and perception) of people with ASD is altered due to experienced stressful life-events. So, for instance, adults with ASD might have experienced stressful auditory exposure to sounds which would let them qualify auditory stimuli as very arousing, even though their physiological response might be similar. However, whether or not ASD adults do indeed physiologically respond more strongly to certain auditory stimuli or whether their auditory sensitivity might be related to experienced stressful life-events are hypotheses that needs further examination.

There are also possible limitations to our study that need to be considered and that could inspire future research. Although all participants in the ASD group scored above the cut-off on the AQ or SRS-A before inclusion, not all scored on the autism spectrum on the ADOS-2. This could indicate that not everyone in our ASD group currently meet ASD criteria. However, given their clinical diagnosis and the recent finding that ADOS-2 module 4 might not be sensitive enough to detect ASD in well-educated autistic adults (e.g. de Bildt et al., 2016; see also for similar findings Lever and Geurts 2018) this seems unlikely. A limitation of our study might be that we do not have ADOS scores from our control group, this means that we cannot rule out the possibility that we could have observed more ASD characteristics in our TD group compared to what they reported in the questionnaires if the ADOS would have been administered. However, their AQ and SRS-A scores were below the cut-off scores for possible ASD and, therefore, did not require further administration of the ADOS. Also, in our study, about 21% of the ASD group and 13% of the TD group were non-responders to our habituation task, which is similar to what has been reported in earlier studies (e.g., Ikezawa et al. 2012; Schell et al. 2002; Schoen et al. 2008). We found that the ASD non-responders showed more ASD symptoms on the ADOS than our ASD responders. In some disorders, such as schizophrenia, non-responders are suggested to be a separate subgroup (e.g., Ikezawa et al. 2012). Perhaps adults with more severe ASD symptomatology are more likely to be a non-responder and indeed form a separate subgroup. Given the heterogeneity of the ASD population, it is plausible that certain subgroups exist. One can imagine that hypo-responsiveness is related to faster habituation and hyper-responsiveness to slower habituation. Although this is an interesting topic, with the current questionnaires available in the Netherlands we were not able to make that distinction between hypo- and hyper-responsiveness. Another possibility is that ASD subgroups with different levels of baseline arousal habituate differently (e.g., Schoen et al. 2008). However, this seems unlikely as our explorative analyses showed no relationship between baseline arousal and habituation or detection threshold. Besides the possibility of subgroups, we did not examine the possible role of predictability and the sense of control over the stimulus (e.g., Robertson et al. 2015). In a neuroimaging study on TD brain responses, an unexpected stimulus can lead to stronger repetition suppression (e.g., Utzerath et al. 2017). Whether or not stimulus-specific expectations also play a role in physiological habituation to auditory stimuli in ASD adults remains unknown. Both subgrouping and the role of expectancy are relevant future research avenues.

In sum, adults with ASD do have more auditory sensitivities and experience certain auditory stimuli as more intense subjectively and possibly physiologically as well (although replication is required). We showed that both physiological habituation and detection thresholds are not likely candidates for underlying mechanisms of auditory sensitivity in adults with ASD.
